# Validity and Reliability of an Artificial Intelligence-Based Posture Estimation Software for Measuring Cervical and Lower-Limb Alignment Versus Radiographic Imaging

**DOI:** 10.3390/diagnostics15111340

**Published:** 2025-05-26

**Authors:** Sung Cheol Park, Sanghee Lee, Jisoo Yoon, Chi-Hyun Choi, Chan Yoon, Yong-Chan Ha

**Affiliations:** 1 Department of Orthopedic Surgery, Seoul Bumin Hospital, Seoul 07590, Republic of Korea; 2 EverEx, Seoul 06134, Republic of Korea; leo@everex.co.kr (C.-H.C.)

**Keywords:** posture estimation, forward head posture, hip–knee–ankle angle, craniovertebral angle, sagittal vertical axis, posture analysis, interrater reliability, cervical alignment, lower-limb alignment

## Abstract

**Background/Objectives**: Accurate postural assessment is essential for managing musculoskeletal disorders; however, routine screening is often limited by radiation exposure, cost, and accessibility constraints of radiography. Recent advances in artificial intelligence (AI) have enabled automated, marker-free analysis using two-dimensional photographs. This study evaluated the validity and reliability of MORA Vu, an AI-based posture estimation software, against radiographic parameters. **Methods**: A prospective pilot study was conducted with 72 participants, divided equally into the cervical and lower-limb alignment groups. Forward head posture (FHP) and digital hip–knee–ankle (DHKA) angles were measured using MORA Vu and compared with corresponding radiographic parameters. Three healthcare professionals independently conducted the AI-based assessments. Correlations were analyzed, and interrater reliability was assessed using the intraclass correlation coefficient (ICC). **Results**: FHP showed the strongest correlation with the craniovertebral angle (r = −0.712) and C2–7 sagittal vertical axis (r = 0.704). The DHKA angle strongly correlated with the radiographic hip–knee–ankle angle (r = 0.754). Interrater reliability demonstrated high agreement (ICC: 0.84 FHP, 0.90 DHKA). **Conclusions**: MORA Vu demonstrated strong validity and high reliability, supporting its potential as a noninvasive screening tool for postural assessment. Given its accessibility and radiation-free nature, it may serve as a viable alternative for routine postural evaluation.

## 1. Introduction

Musculoskeletal disorders (MSDs) are a major cause of severe long-term pain and physical disability worldwide [1,2,3]. They affect approximately 25–30% of the general population [1,3,4]. Body alignment, considered a crucial health indicator, has been identified as a significant factor in the development of MSDs [5,6,7]. Postural malalignment may be associated with acute and chronic pain, gait abnormalities, decreased activities of daily living, and compromised physical and psychological well-being [8,9]. Consequently, the implementation of accessible and reliable assessment methods for postural malalignment is a critical priority in both clinical practice and public health settings.

Reliable assessments of body alignment may enable the monitoring of postural changes over time and early detection of abnormalities, facilitating timely and appropriate interventions [8]. Plain radiography serves as the gold standard diagnostic tool for postural evaluation, offering cost-effectiveness, easy availability, and rapid operability [10,11]. However, this may involve the risk of radiation exposure, substantial equipment costs, and professional skills to operate, making periodic evaluations impractical [12,13].

Hence, various noninvasive methods for evaluating body alignment have been suggested as alternatives, eliminating the risk of radiation exposure. Photogrammetry, which involves measuring objects on photographs, represents a viable, cost-effective, and noninvasive alternative compared to plain radiography, while providing greater objectivity than visual assessment [14]. However, traditional photogrammetric methods often rely on examiner-dependent processes, including the manual placement of physical markers or manual annotation of anatomical landmarks on images [5,8,14,15,16,17]. Some studies have explored markerless systems using dedicated hardware or computer software [9,18], yet their integration into everyday clinical settings remains limited by cost and equipment requirements.

Recent advances in artificial intelligence (AI)-based pose estimation technology now enable the automatic detection of anatomical keypoints from standard photographs without requiring physical markers or manual labeling. In this study, we evaluated an AI-based posture estimation software, MORA Vu, which calculates alignment parameters using only photographs taken with mobile devices such as smartphones or tablets. The software automatically identifies 24 anatomical reference points and calculates postural angles—most notably the forward head posture (FHP) angle and the digital hip–knee–ankle (DHKA) angle—without requiring real-world calibration or specialized hardware.

Compared to previous research on photogrammetry or motion analysis, our study provides novel clinical validation of a fully automated, markerless posture estimation system by comparing its results with radiographic gold standards. To our knowledge, few studies have directly assessed the agreement between AI-derived postural measurements and radiographic references in symptomatic patients. Therefore, this study aimed to assess the clinical validity and reliability of an AI-based posture estimation software that operates on mobile devices by comparing its automatically derived alignment measurements with conventional radiographic parameters in patients with cervical or knee symptoms.

## 2. Materials and Methods

### 2.1. Study Design and Participants

This study was a prospective pilot investigation approved by the Institutional Review Board of Bumin Hospital, Seoul (IRB no BMH 2024-06-016; approval date: 26 June 2024). Participants were recruited from an orthopedic outpatient clinic between August and November 2024. Two separate groups were recruited for the evaluation of cervical and lower-limb alignment, with each group comprising 36 participants. The sample size was determined with reference to a previous study that compared musculoskeletal joint angles measured using mobile devices and radiographic imaging, which reported significant correlations based on a sample of 31 participants [13]. To account for potential dropouts and ensure statistical validity, we enrolled 36 participants in each group.

The inclusion criteria for the cervical alignment group were individuals reporting pain or alignment abnormalities in the cervical spine, whereas the lower-limb alignment group included participants with knee joint pain or alignment abnormalities. The common inclusion criteria for both groups were women and men aged ≥19 years. Individuals with severe musculoskeletal pain or disorders that hindered their ability to maintain a static posture during the evaluation were excluded.

### 2.2. Body Alignment Assessment Using an AI-Based Posture Estimation Software

All participants underwent measurements using an AI-based posture estimation software (MORA Vu, Ver 1.2.0). MORA Vu is a musculoskeletal analysis software device authorized for medical use that employs convolutional neural networks and multilayer perceptron algorithms to identify 24 anatomical reference points from digital photographs and calculate the angles formed between these points. The software was used in its original commercially available form without any retraining or modifications. Digital photographs were captured from a distance of 3 m using a sixth-generation iPad Air 11 mounted on a tripod. The tripod was positioned at a distance of 3 m and a height of 1.2 m relative to the participant to ensure proper horizontal alignment.

All MORA Vu assessments were independently conducted by three healthcare professionals who were blinded to the radiographic results. To assess interrater reliability, each participant was photographed once by each examiner, resulting in three images per participant. For each image acquisition, the healthcare professional independently repositioned the tripod, camera, and participant to ensure examiner independence and minimize systematic bias.

Following image capture, the software automatically identified anatomical keypoints on each image and performed angle computations. Importantly, MORA Vu calculates angles directly from the two-dimensional pixel coordinates of anatomical keypoints without applying any geometric corrections or converting pixel values into real-world distances. This method ensures consistency and eliminates the need for manual intervention, as all measurements were fully automated and completed within seconds.

For cervical alignment assessment, the MORA Vu measured the FHP angle from lateral photographs. This angle was calculated as the angle formed between a vertical reference line and a line connecting the center of C7 and the center of the head, both of which were anatomical reference points identified using the AI-based posture estimation software.

**Figure 1:**
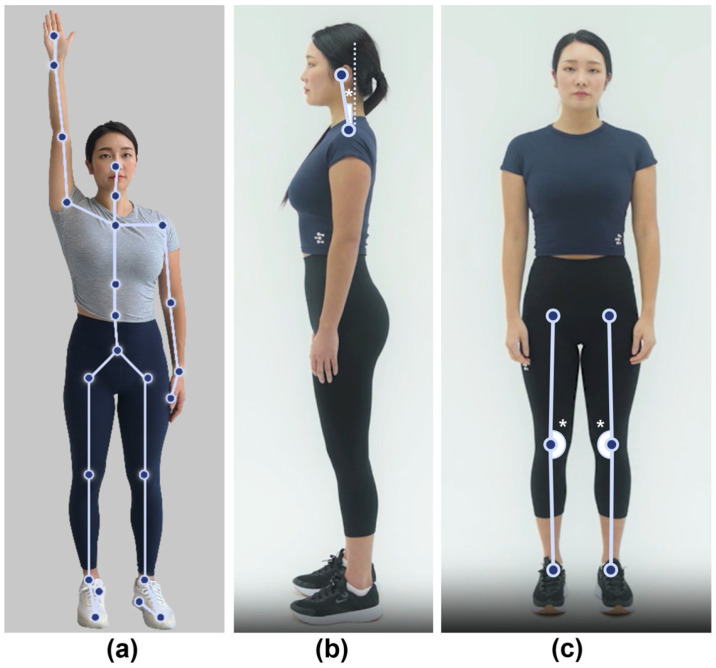
Body alignment assessment using an AI-based posture estimation software. (**a**) Anatomical key reference points detected by MORA Vu. (**b**) Forward head posture angle. (**c**) Digital hip–knee–ankle angle. * Indicates the locations where the angles shown in (**b**,**c**) were measured on the body image.

To assess lower-limb alignment, we used the MORA Vu to measure the DHKA angle using frontal photographs. The software identified the line connecting the hip joint center to the knee joint center and the line connecting the center of the knee joint to the center of the ankle joint. The angle between the two lines was then calculated. To minimize variability, we standardized the distance between the heels to 20 cm during both the MORA Vu and radiographic measurements. Figure 1 shows the MORA Vu body alignment measurements.

### 2.3. Radiographic Measurements

Radiographic measurements of cervical alignment were independently performed by an orthopedic spine specialist, while lower-limb alignment was assessed by a separate orthopedic surgeon specializing in knee surgery. Both evaluators were blinded to the AI-based measurements.

Cervical alignment was assessed using standing position lateral radiographs of the cervical spine. The parameters of cervical tilt, cranial tilt, C7 slope, T1 slope, C2–7 Cobb angle, craniovertebral angle (CVA), and C2–7 sagittal vertical axis (SVA) were evaluated by a single orthopedic spine specialist [19,20,21]. These parameters are defined in Table 1.

**Figure 2:**
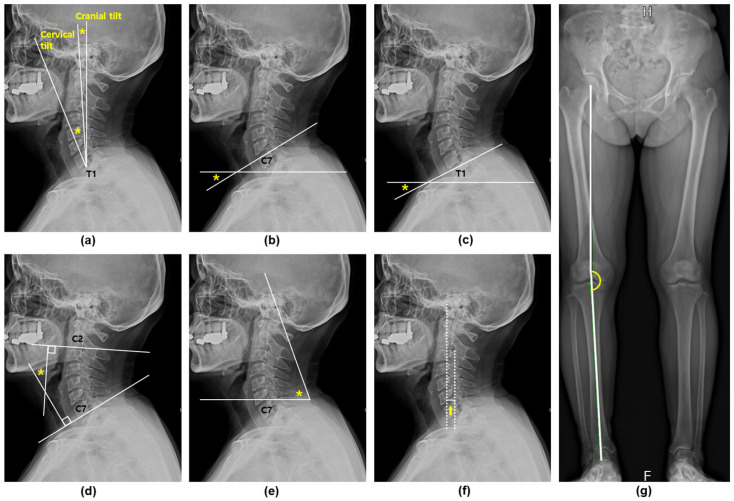
Radiographic assessment of cervical spine and lower-limb alignment using cervical lateral X-ray and EOS anteroposterior radiographs. (**a**) Cervical tilt and cranial tilt. (**b**) C7 slope. (**c**) T1 slope. (**d**) C2–7 Cobb angle. (**e**) Craniovertebral angle. (**f**) C2–7 sagittal vertical axis. (**g**) Radiographic hip–knee–ankle angle. * Indicates the locations where the angles shown in (**a**–**e**) were measured on the radiographs. The arrow indicates the C2–7 sagittal vertical axis in panel (**f**).

To assess the lower-limb alignment, radiographic imaging was performed using EOS anteroposterior radiographs (EOS Imaging, Paris, France). The radiographic hip–knee–ankle (RHKA) angle was defined as the angle formed by the mechanical axes of the femur and tibia. In a neutrally aligned knee, this line passes through the femoral head center, the tibial intercondylar eminence midpoint, and the talus center. Cervical spine and lower-limb alignment measurements from the radiographs are shown in Figure 2.

### 2.4. Statistical Analysis

All statistical analyses were performed using R Studio (version 4.1.2), and statistical significance was set at *p* < 0.05. The validity of MORA Vu measurements was assessed through correlation analyses comparing the FHP angles measured by MORA Vu with cervical alignment parameters obtained from plain radiographs, as well as the DHKA angles measured by MORA Vu with the RHKA angles measured from EOS imaging. Normality of data distribution was evaluated using the Shapiro–Wilk test. For FHP measurements, the data did not follow a normal distribution; thus, Spearman’s rank correlation coefficient was applied for all analyses involving FHP angles and cervical alignment parameters. Conversely, the DHKA–RHKA angle comparison followed a normal distribution; therefore, Pearson’s correlation coefficient was applied in this analysis. The mean values of the FHP and DHKA angles derived from measurements by three independent evaluators were used for correlation analyses to ensure consistency across evaluators.

The interrater reliability of the MORA Vu measurements was evaluated for both FHP and DHKA angles using the intraclass correlation coefficient (ICC). Given that three fixed healthcare professionals independently evaluated all participants, a two-way mixed-effects model with absolute agreement and single measures (ICC [3,1]) was applied, as described by Shrout and Fleiss (1979) [22]. The ICC was calculated using the formula: ICC (3,1) = (MS_B_ − MS_E_)/[MS_B_ + (*k* − 1)MS_E_], where MS_B_ represents the mean square between subjects, MS_E_ denotes the residual mean square (error), and *k* indicates the number of raters.

In addition, to evaluate the agreement between the DHKA and RHKA angles at the individual level, a Bland–Altman analysis was performed. The mean difference (bias) and 95% limits of agreement were computed to assess the degree of measurement interchangeability between the AI-based and radiographic methods, providing further insight into their clinical comparability.

## 3. Results

### 3.1. Participant Characteristics

A total of 72 participants were enrolled in this study, with 36 participants allocated to each of the cervical and lower-limb alignment evaluation groups. The cervical alignment evaluation group comprised 11 men (31%) and 25 women (69%), with a mean age of 46.8 ± 14.2 years. The lower-limb alignment evaluation group included 14 men (39%) and 22 women (61%), with a mean age of 57.1 ± 15.1 years.

### 3.2. Alignment Measurements

Table 2 presents the descriptive statistics of the alignment measurements using the AI-based posture estimation software. The mean FHP angle was 15.1 ± 4.1°, and the mean DHKA angle was 177.3 ± 2.9°.

Table 3 shows the alignment measurements obtained from cervical lateral radiographs and EOS anteroposterior radiographs. The mean cervical and cranial tilts were 16.7° and 7.7°, respectively. The mean T1 and C7 slopes were 23.2° and 20.9°, respectively. The CVA and C2–7 SVA were measured at 63.7° and 2.1 cm, respectively. Regarding lower-limb alignment, the mean RHKA angle was 178.2°.

### 3.3. Correlation Between MORA Vu and Radiographic Measurements

Table 4 presents the correlation analysis between the MORA Vu measurements and radiographic alignment parameters. The FHP demonstrated the strongest negative correlation with CVA (r = −0.712, *p* < 0.001) and a strong positive correlation with C2–7 SVA (r = 0.704, *p* < 0.001). For lower-limb alignment, the DHKA angle was strongly correlated with the RHKA angle (r = 0.754, *p* < 0.001).

**Figure 3:**
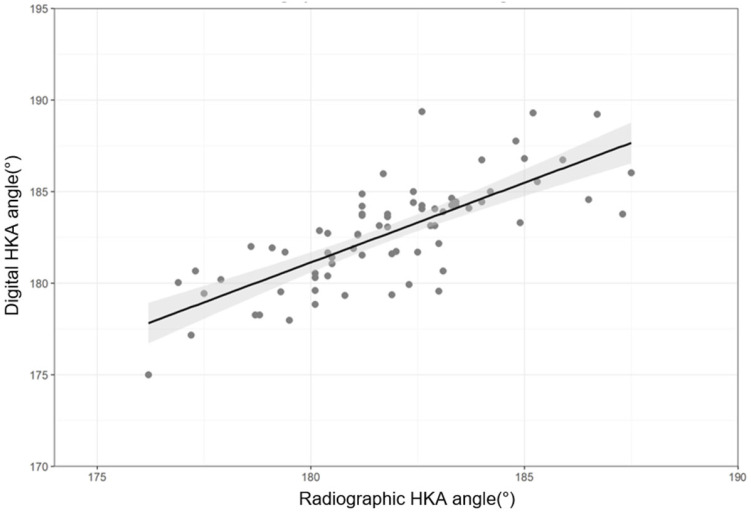
Scatter plot depicting the correlation between the digital hip-knee-ankle angle measured by the MORA Vu software and the radiographic hip-knee-ankle angle (r = 0.754, *p* < 0.001). The solid line indicates the linear regression fit, and the shaded area represents the 95% confidence interval.

Figure 3 presents a scatter plot demonstrating a strong positive correlation between the AI-derived DHKA angle and the RHKA angle. To further evaluate agreement at the individual level, a Bland–Altman analysis was also conducted, revealing a small mean bias of 0.89° (standard deviation of differences 1.90°) with 95% limits of agreement ranging from −2.83° to 4.62° (Supplementary Figure S1).

### 3.4. Interrater Reliability of AI-Based Posture Estimation Software Measurements

The ICCs for FHP and DHKA angles were 0.84 and 0.90, respectively, indicating high reliability for both cervical and lower-limb alignment measurements (Table 5).

## 4. Discussion

We propose an AI-based, noninvasive posture estimation software for evaluating cervical spine alignment in the sagittal plane and lower-limb alignment in the coronal plane as a novel approach to body posture assessment. Statistically significant correlations were identified between the measurements obtained using this software and the traditional radiographic parameters. Furthermore, alignment measurements using the software demonstrated excellent interrater reliability for both the cervical spine and lower limb assessments.

The MORA Vu system employs advanced AI algorithms for the automated identification of anatomical reference points in mobile camera images. By analyzing the two-dimensional coordinates of these points, the system calculates the angles formed between specific anatomical landmarks and provides detailed measurements of joint alignment and spinal curvature. This radiation-free and noninvasive system offers several potential advantages in clinical settings, including cost-effectiveness due to mobile device compatibility and the possibility of application in community-based or semi-supervised screening environments, without the need for specialized hardware or physical markers. Given its high accessibility, it could be an effective screening tool for alignment.

Previous studies have reported that approximately 70% of the population experiences neck pain [23]. An FHP relative to the trunk in the sagittal plane has been reported as one of the primary causes of neck pain [18,24,25]. The increasing use of smartphones and computers has accelerated the occurrence of FHP, leading to growing attention being paid to this condition. Consistent with previous reports on the assessment of cervical alignment using digital photographs, our findings showed significant correlations between the values measured by the investigated device or software and radiographic parameters [17,18].

In this study, we employed the FHP angle as a postural assessment metric, utilizing the center of the head and C7 vertebral body as reference points. This approach differs from those of previous studies that predominantly used CVA or forward neck tilt angle measured between the tragus of the ear and the shoulder [18]. Nevertheless, the assumption that the tragus approximates the center of the head can be considered reasonable [26], suggesting that our postural assessment metric shares fundamental alignment principles with previously proposed measures. Furthermore, no clear consensus has been established regarding standardized assessment metrics and diagnostic criteria for FHP. Further systematic research is needed to establish standardized postural assessment metrics and improve communication in research and clinical fields regarding FHP evaluation.

Although the FHP angle measured by this software showed strong correlations with key radiographic parameters such as cranial tilt, CVA, and C2–7 SVA [17,20,27], it should be noted that each of these parameters provides complementary information relevant to cervical sagittal balance. Therefore, while our findings support the potential of AI-based posture estimation software as a radiation-free screening tool, its clinical interpretation should be made in conjunction with broader biomechanical and symptomatic contexts.

The hip–knee–ankle (HKA) angle is a widely established method for evaluating lower-limb alignment [28]. Previous studies have reported that HKA angle malalignment is associated with the incidence and progression of knee osteoarthritis (OA) and the prognosis of surgical interventions [28,29]. Consequently, the HKA angle assessment plays a crucial role in the management of knee OA. However, full-length standing lower-limb radiography, the primary assessment method, has several limitations, including increased radiation exposure, the requirement for specialized technician training, and prolonged image acquisition time [28].

The DHKA angle measurements obtained using the MORA Vu software demonstrated a strong correlation with radiographic values and high interrater reliability, suggesting its potential clinical utility in evaluating lower-limb alignment. To further assess its clinical interchangeability with radiography, we performed a Bland–Altman analysis comparing DHKA and RHKA angles. Despite nonsimultaneous acquisition—conducted with consistent foot positioning but at separate time points—the analysis revealed a small mean bias of 0.89° and a standard deviation of 1.90°, indicating good agreement at the individual level. Notably, these findings are comparable to those of Saiki et al., who used simultaneous image acquisition with an OpenPose-based two-dimensional (2D) deep learning system and reported a bias of −1.08° with a standard deviation of 2.17° [28]. The fact that MORA Vu achieved similar agreement without the advantages of simultaneous acquisition underscores the robustness and real-world applicability of our approach.

This study had some limitations. First, the relatively small sample size limits the generalizability of our results, necessitating larger, well-designed studies to validate our findings. Second, the reference markers identified by the AI-based software may not precisely correspond to the traditional anatomical landmarks used in previous studies on postural assessment metrics and radiographic measurements. Lastly, the nonsimultaneous acquisition of the MORA Vu evaluation and radiographic imaging could potentially introduce measurement discrepancies. Nevertheless, we implemented a standardized positioning protocol to ensure consistent patient posture during both assessments. Moreover, the study design and sample distribution did not permit a comprehensive evaluation of diagnostic accuracy using binary classification metrics, such as sensitivity, specificity, or predictive values. As such, although the software shows promise as a screening tool, future research with larger and more diverse cohorts is required to formally validate its diagnostic performance.

Despite these limitations, to the best of our knowledge, this is the first analysis of cervical spine and lower-limb alignment using an AI-based, noninvasive posture estimation software that operates on mobile devices and automatically designates reference points. This novel approach could be a screening tool for postural alignment abnormalities in daily life. Additionally, it may be useful for assessing treatment outcomes and prognosis, further broadening its clinical applicability. Furthermore, the clinical utility of this technology may be enhanced by expanding its application to other musculoskeletal conditions, suggesting promising prospects for its broader implementation in clinical practice.

## 5. Conclusions

This study demonstrated that an AI-based, noninvasive posture estimation software exhibited strong correlations with radiographic measurements and high interrater reliability in assessing cervical spine and lower-limb alignment. Its key innovation lies in the fully automated calculation of alignment angles from 2D photographs without requiring physical markers or manual annotation. Given its accessibility, ease of use, and radiation-free nature, this method may serve as a practical screening tool in both clinical and public health contexts. Further research involving larger and more diverse populations is needed to confirm the clinical utility, reproducibility, and broader applicability of this approach.

## Figures and Tables

**Table 1:** Cervical alignment parameters.

Parameter	Description
Cervical tilt	The angle between the vertical line from the center of the T1 upper endplate (T1UEP) and the line from the center of T1UEP to the tip of the dens
Cranial tilt	The angle between the line from the center of the T1UEP to the dens and the sagittal vertical axis from the T1UEP
C7 slope	The angle between the C7 upper endplate line and the horizontal plane
T1 slope	The angle between the T1UEP line and the horizontal plane
C2–7 Cobb angle	The angle formed by the intersection of perpendicular lines from the lines parallel to the lower endplates of C2 and C7
Craniovertebral angle	The angle between the horizontal line and the line from the distal tip of the C7 spinous process to the external auditory canal
C2–7 sagittal vertical axis	The distance between the plumb line from the center of C2 and the posterosuperior corner of the C7 vertebral body

**Table 2:** Alignment measurements by AI-based posture estimation software.

Parameter	Evaluator A	Evaluator B	Evaluator C	Overall
FHP angles (°)	15.1 ± 4.3	15.1 ± 3.6	15.1 ± 4.3	15.1 ± 4.1
DHKA angles (°)	177.3 ± 2.9	177.2 ± 3.0	177.4 ± 3.0	177.3 ± 2.9

Data are shown as means ± standard deviations. AI, artificial intelligence; FHP, forward head posture; DHKA, digital hip–knee–ankle.

**Table 3:** Alignment measurements from radiographs.

Parameter	Values
Cervical tilt (°)	16.7 ± 5.7
Cranial tilt (°)	7.7 ± 4.8
T1 slope (°)	23.2 ± 6.8
C7 slope (°)	20.9 ± 7.4
C2–7 Cobb angle (°)	11.9 ± 9.7
CVA (°)	63.7 ± 5.4
C2–7 SVA (cm)	2.1 ± 1.0
RHKA angle (°)	178.2 ± 2.5

Data are shown as means ± standard deviations. CVA, craniovertebral angle; SVA, sagittal vertical axis; RHKA, radiographic hip–knee–ankle.

**Table 4:** Correlation analysis between MORA Vu and radiograph-based measurements.

Comparison	Correlation Coefficient (r)	*p*-Value
FHP angle vs. cervical tilt	−0.048	0.783
FHP angle vs. cranial tilt	0.611	<0.001
FHP angle vs. T1 slope	0.417	0.011
FHP angle vs. C7 slope	0.424	0.010
FHP angle vs. C2–7 Cobb angle	0.032	0.852
FHP angle vs. CVA	−0.712	<0.001
FHP angle vs. C2–7 SVA	0.704	<0.001
DHKA angle vs. RHKA angle	0.754	<0.001

FHP, forward head posture; CVA, craniovertebral angle; SVA, sagittal vertical axis; DHKA, digital hip–knee–ankle; RHKA, radiographic hip–knee–ankle.

**Table 5:** Interrater reliability of MORA Vu measurements.

Parameter	ICC
FHP angle	0.84
DHKA angle	0.90

ICC, intraclass correlation coefficient; FHP, forward head posture; DHKA, digital hip–knee–ankle.

## Data Availability

The data supporting the findings of this study are available from the corresponding author upon reasonable request and are subject to ethical approval and confidentiality agreements.

## Supplementary Materials

The following supporting information can be downloaded at https://www.mdpi.com/article/10.3390/diagnostics15111340/s1: Supplementary Figure S1. Bland–Altman plot assessing the agreement between digital and radiographic hip-knee-ankle angles. The solid line indicates the mean difference, and the dashed lines represent the 95% limits of agreement.

## Abbreviations

The following abbreviations are used in this manuscript:

AI	artificial intelligence
CVA	craniovertebral angle
DHKA	digital hip–knee–ankle
FHP	forward head posture
ICC	intraclass correlation coefficient
MSDs	musculoskeletal disorders
OA	osteoarthritis
RHKA	radiographic hip–knee–ankle
SVA	sagittal vertical axis
