# Supplementary figures and images for: Validity and Reliability of an Artificial Intelligence-Based Posture Estimation Software for Measuring Cervical and Lower-Limb Alignment Versus Radiographic Imaging
Source: Diagnostics (Basel). 2025 May 26;15(11):1340. doi: 10.3390/diagnostics15111340 (PMC12155411; doi:10.3390/diagnostics15111340)

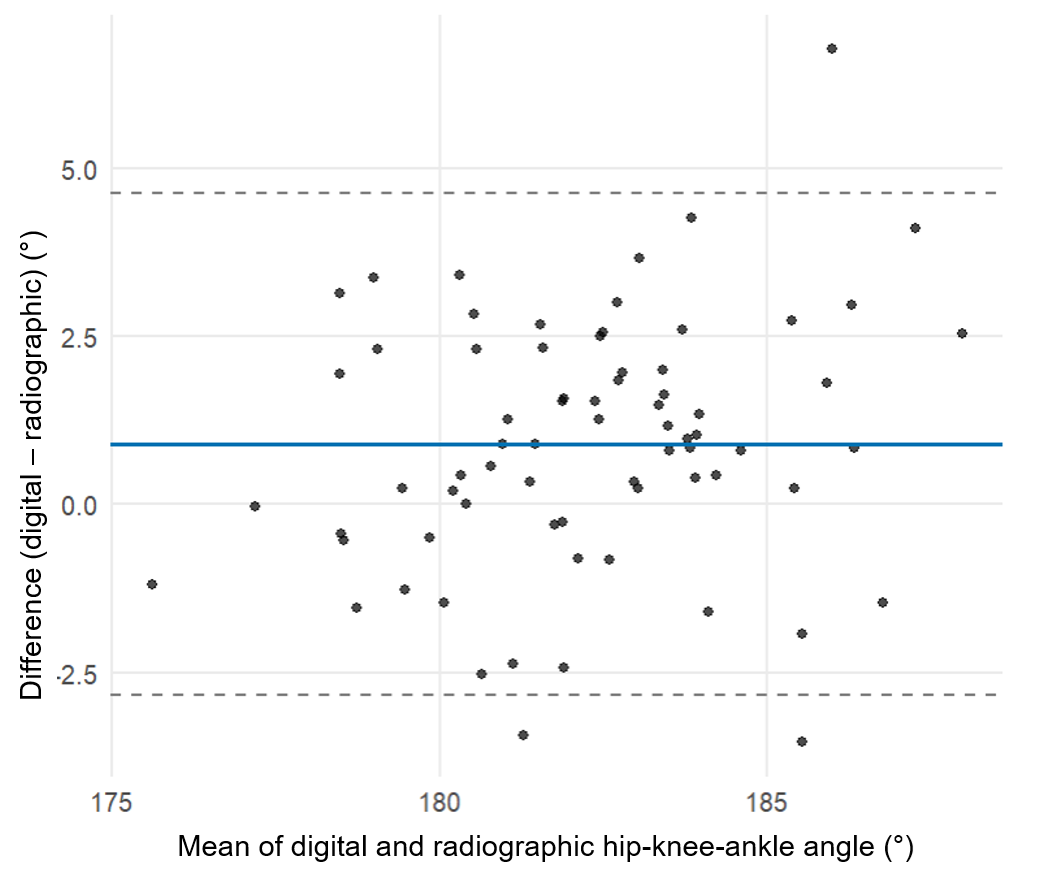

Supplement: Supplementary file 1 [file diagnostics-15-01340-s001.zip › diagnostics-3595331-supplementary Figure S1.png]
